# Imaging in children with ataxia-telangiectasia—The radiologist’s approach

**DOI:** 10.3389/fped.2022.988645

**Published:** 2022-09-16

**Authors:** Katarzyna Jończyk-Potoczna, Jakub Potoczny, Aleksandra Szczawińska-Popłonyk

**Affiliations:** ^1^Department of Pediatric Radiology, Institute of Pediatrics, Pozna University of Medical Sciences, Poznań, Poland; ^2^Department of Radiology, Greater Poland Cancer Center, Poznań, Poland; ^3^Department of Pediatric Pneumonology, Allergy and Clinical Immunology, Institute of Pediatrics, Poznań University of Medical Sciences, Poznań, Poland

**Keywords:** imaging, ataxia-telangiectasia, magnetic resonance, ultrasound, lymphadenopathy, children

## Abstract

Ataxia-telangiectasia (A-T) is a syndromic inborn error of immunity (IEI) characterized by genomic instability, defective reparation of the DNA double-strand breaks, and hypersensitivity to ionizing radiation disturbing cellular homeostasis. The role of imaging diagnostics and the conscious choice of safe and advantageous imaging technique, as well as its correct interpretation, are crucial in the diagnostic process and monitoring of children with A-T. This study aimed at defining the role of a radiologist in the early diagnosis of A-T, as well as in detecting and tracking disease complications associated with infections, inflammation, lymphoproliferation, organ-specific immunopathology, and malignancy. Based on our single-center experience, retrospective analysis of investigations using ionizing radiation-free techniques, ultrasound (US), and Magnetic Resonance Imaging (MRI), was performed on regularly followed-up 11 pediatric A-T patients, 6 girls and 5 boys, aged from 2 to 18 years, with the longest period of observation coming to over 13 years. Our attention was especially drawn to the abnormalities that were observed in the US and MRI examinations of the lungs, abdominal cavity, and lymph nodes. The abdominal US showed no abnormalities in organ dimensions or echostructure in 4 out of 11 children studied, yet in the other 7, during follow-up examinations, hepato- and/or splenomegaly, mesenteric, visceral, and paraaortic lymphadenopathy were observable. In 2 patients, focal changes in the liver and spleen were shown, and in one patient progressive abdominal lymphadenopathy corresponded with the diagnosis of non-Hodgkin lymphoma (NHL). The lung US revealed multiple subpleural consolidations and B line artifacts related to the interstitial-alveolar syndrome in 5 patients, accompanied by pleural effusion in one of them. The MRI investigation of the lung enabled the detection of lymphatic nodal masses in the mediastinum, with concomitant airway lesions characteristic of bronchiectasis and focal parenchymal consolidations in one A-T patient with chronic respiratory failure. This patient also manifested organomegaly and granulomatous liver disease in abdominal MRI examination. Our study shows that the use of modern US capabilities and MRI is safe and efficient, thereby serving as a recommended advantageous imaging diagnostic tool in monitoring children with IEI and DNA instability syndromes.

## Introduction

Ataxia-telangiectasia (A-T) is a syndromic inborn error of immunity (IEI) characterized by genomic instability, defective response to genotoxic factors, impaired reparation of the DNA double-strand breaks, and hypersensitivity to ionizing radiation. A dysregulation of ataxia-telangiectasia mutated (ATM) protein kinase nuclear and cellular functions underpinning the pathophysiology of the disease are related to the defective generation of reactive oxygen species, mitochondrial dysfunctions, alterations in transcription and splicing, and impaired cellular protein homeostasis (1, 2). The multiplicity of ATM nuclear and cellular activities maintaining homeostasis are reflected in the constellation of phenotypic features of A-T.

A-T is a complex, multisystemic disease at the interface of immunodeficiency, infections, autoimmunity, autoinflammation, lymphoproliferation, and malignancy. The leading A-T symptomatology is characterized by neurodegeneration and progressively debilitating cerebellar ataxia with postural instability, oculomotor apraxia, dysarthria, and orolingual insufficiency, as well as extrapyramidal dysfunctions with choreoathetotic movements, dystonia, and muscle tremor. Affected children suffer from chronic rhinosinusitis, obstructive airway disease, bronchiectasis, and interstitial lung disease or pneumonia with fibrosis. The respiratory disease is exacerbated by dysfunctional swallowing, gastroesophageal reflux, aspiration episodes, and ineffective coughing (3, 4). An impaired response to oxidative stress may have a causal relationship with chronic non-alcoholic fatty liver disease in pediatric patients with A-T (5, 6). The extended A-T phenotype also includes hormonal dysfunctions, such as growth hormone deficiency, gonadal failure, and diabetes (7, 8), cutaneous and systemic laryngeal, pulmonary, and hepatosplenic granulomatosis (9–11). A-T is, therefore, a multisystemic devastating disease, burdened with a high rate of malignant transformation, significantly reducing life expectancy (12). While the monitoring of the clinical course of A-T in children relies on a multidisciplinary team of a pediatrician, neurologist, endocrinologist, pulmonologist, gastroenterologist, and hematologist under the pediatric immunologist’s supervision (13, 14), the radiologist’s role in diagnosing and following-up of affected patients cannot be overestimated. The A-T patient group is affected with a severe IEI with susceptibility to ionizing radiation, thus the choice of an adequate, safe, and providing best answer to the clinician’s requests imaging diagnostic technique is crucial for the patient’s monitoring.

According to the expert guidelines (13, 15, 16) application of ionizing radiation-free imaging techniques for the assessment of the respiratory tract should be accompanied by regular lung function monitoring aimed at early detection of lung disease progression and indicating the need for therapeutic interventions.

In this study, we aimed at defining the role of a radiologist in the early diagnosis of A-T, as well as in detecting and tracking disease complications associated with infections, inflammation, lymphoproliferation, organ-specific immunopathology, and malignancy. Based on our single-center experience, we also tried to find out whether investigations using ionizing radiation-free techniques, ultrasound (US), and Magnetic Resonance Imaging (MRI), as these techniques require no ionizing radiation and therefore, can be an elegant alternative to X-ray or CT imaging.

## Patients and methods

We retrospectively reviewed medical records of 11 children with A-T, 6 girls and 5 boys, aged from 2 to 18 years, who had been diagnosed and treated in our university pediatric tertiary care center. All the children studied were regularly monitored in the pediatric immunology unit and simultaneously, followed up in the pediatric radiology department. The mean age of the definitive A-T diagnosis, the age of the first diagnostic imaging examination, the technique that had been used, as well as the time of observation, were investigated. An in-depth analysis of findings observable in imaging of the lungs, abdominal cavity, lymph nodes, and due to special clinical indications, also in the neck with larynx, and joints was performed. The US and MRI examinations were performed during follow-up visits and additionally, during exacerbations of chronic symptoms or acute pathology.

The basic US examination protocol included the lungs (transthoracic US), peripheral lymph nodes, and abdominal cavity, with the latter supplied by analysis of vascular flow in Color Doppler (CD). Currently, superb microvascular imaging (SMI) which enables visualization of low microvascular flow, as well as elastography using low-frequency vibrations to show elasticity/stiffness of the organ, are also included in the protocol of the US examination of the abdominal cavity. The US of the abdominal cavity and peripheral lymph nodes were conducted in a supine position, while the lung US was done in a sitting patient.

The chest US examination protocol was implemented according to the following standard: the examination was performed using a linear probe of 5–12 mHz (L12-5) frequency and, depending on the patient’s age, with either a convex probe of 1–5 mHz (C5-1) frequency, a convex probe of 4–9 mHz (C9-4) frequency or a microconvex probe of 5–8 mHz (C8-5) frequency through longitudinal and transverse sections of the anterior, lateral, and posterior walls of the chest. The preliminary preset was soft tissue, excluding artifact reduction options (SonoCT, XRes). Doppler imaging was used for the evaluation of vascularization of the inflammatory changes. The chest US examination was performed by applying the probe to the anterior, lateral, and posterior surfaces of the chest. Transverse sections of the chest wall were obtained by the transverse application of the probe and scanning the whole available area in the craniocaudal direction. Longitudinal sections were obtained by applying the probe along the parasternal line, the midclavicular line, the anterior axillary line, the midaxillary line, the posterior axillary line, the scapular line, and the paravertebral line moving the probe along the intercostal spaces. In every patient the following elements were evaluated: the quality (free flowing or organized, localization) and quantity (fluid layer in millimeters) of any fluid present in the pleural space, the shape and thickness of the pleural line, the lung sliding sign, A-lines and B-lines artifacts (their number, localization, and morphology, including single ones as well as “lung rockets” complexes and “white lung” images) and alveolar consolidations (their number, dimensions, localization, morphology, presence of bronchogram and its characteristic (air or fluid) and vascularization).

The diagnostic monitoring protocol also included MRI in A-T patients to monitor the liver and spleen pathology. This technique allows to achieve high resolution and precision of focal lesions analysis due to diffusion-weighted and susceptibility-weighted imaging (DWI and SWI, respectively) or fat saturation sequences. The chest MRI protocol implemented in the patients studied has been displayed in Table 1.

**TABLE 1:** The chest magnetic resonance imaging (MRI) protocol used in the patients studied.

The chest MRI protocol		
Sequence	Orientation	Contrast medium
T2 Haste	Cor	Native
T2 Haste	Tra	Native
T1 Vibe	Cor	Native
T1 Vibe	Tra	Native
T2 blade	Cor	Native
T2 Blade	Tra	Native
T1 Twist	Cor	Dynamic
T1 Vibe	Cor	Post-contrast
T1 Vibe	Tra	Post-contrast

## Results

### Demographic data

Eleven pediatric patients with a genetically confirmed diagnosis of A-T, aged from 2 to 18 years, were included in the study group. The mean age of initial diagnosis of A-T was between 3 and 4 years. All the patients studied had the classical form of A-T with the early onset disease as defined by Driessen et al. (17), neurodegeneration and progressive cerebellar ataxia, severe antibody deficiency with impaired B- and T-cell homeostasis, remarkably increased alpha-fetoprotein (AFP) levels and recurrent respiratory tract infections. Besides infectious complications, immune dysregulation in the form of autoimmunity was also a frequent sequela among the A-T children studied, with autoimmune hemolytic anemia diagnosed in 4 out of 11 of them. Malignancy, in the form of non-Hodgkin lymphoma (NHL), was diagnosed in one patient. Two patients deceased due to liver failure and a chronic Epstein–Barr virus (EBV) infection. Whereas hypogammaglobulinemia, with low serum IgG and IgG2 levels in 9 out of 11 patients, immunoglobulin replacement therapy (Ig-RT) has been implemented, either as a hospital-based intravenous (IVIg) or home-based subcutaneous (SCIg) treatment. The youngest participant had his first diagnostic imaging examination 5 months before the final diagnosis was established. The longest period of observation in one patient was 13 years, from the diagnosis of A-T at the age of 5 years to his death. Other A-T patients have been monitored in our pediatric radiology department for a period from 2 to 10 years. The basic demographic data of the A-T children studied are displayed in Table 2.

**TABLE 2:** Demographic data of ataxia-telangiectasia (A-T) patients and the time of observation by a pediatric radiologist.

Demographic data of A-T patients			

Patient’s number	Gender	Patient’s age at diagnosis (years)	Time of observation at report (years)
Pt 1	M	3	2
Pt 2	F	2	6
Pt 3	M	2	3
Pt 4	F	3	2
Pt 5	F	7	10
Pt 6	M	6	13
Pt 7	F	2	2
Pt 8	F	3	2
Pt 9	F	2	4
Pt 10	M	8	10
Pt 11	M	10	2

### Ultrasound assessment

All participants of the study underwent an abdominal US examination. The most common reasons for the referral were searching for the infection outbreak site, and non-infectious disease sequelae, such as lymphoproliferation, lymphadenopathy, liver disease, and granulomatosis. In only 4 patients observed over time, there were no abnormalities in terms of organ measurements and their echostructure. In the other 7 patients, the sonographic image of the abdomen was normal in the early stage of the disease. Then, during follow-up examinations, hepato- or splenomegaly as well as mesenteric, visceral, and paraaortic lymph node enlargement was diagnosed. Besides, in two patients, focal changes in the liver and spleen were observed.

**FIGURE 1:**
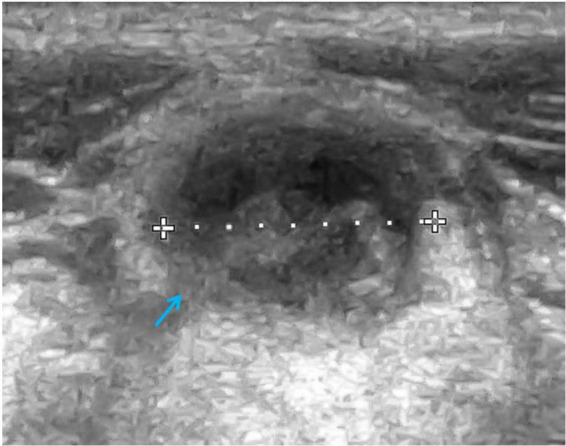
Ultrasound (US) examination of the lymph node, linear probe. A cervical lymph node with an abnormal echostructure, heterogeneous hypoechogenicity, and change of a shape from oval to round (marked with an arrow).

Cervical, submandibular, and supraclavicular lymph nodes size and echostructure were also investigated and abnormalities were detected in 5 patients. In one female patient, in whom lymphadenopathy in the form of pathological lymph node echostructure with dense hilum, decreased echogenicity and change of shape were described, and in the follow-up examination enlargement of mediastinal and supraclavicular lymph nodes was revealed, the diagnosis of NHL was established. Other abnormalities in A-T patients included a change of lymph node shape from oval to round and decreased echogenicity. The pathological transformation of a cervical lymph node which requires a pediatrician’s awareness due to its possible malignant, infectious, or immunological etiology and thus needs further diagnostic procedures, is shown in Figure 1. No pathological vascular flow was diagnosed in the CD examination, apart from one case, in which a hilum overlay was accompanied by abnormal peripheral flow in the lymph nodes.

**FIGURE 2:**
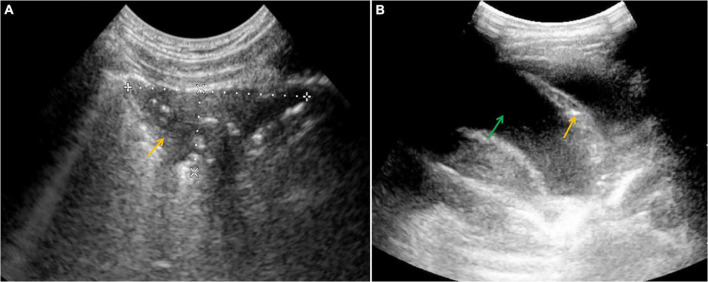
Ultrasound (US) examination of the lung, convex probe, **(A)** an area of subpleural consolidation (marked with an arrow), **(B)** pleural effusion (a green arrow shows the fluid and an orange one shows atelectasis).

In 5 out of our 11 A-T patients who underwent lung US examination, multiple, partially merging subpleural consolidations and numerous B line artifacts were detected, corresponding with the interstitial-alveolar syndrome. In two cases, pleural effusion was found. The lung US images of consolidations and pleural effusion are shown in Figures 2A,B, respectively. The summary of the abdominal, lymph node, and lung US findings detected and monitored in the A-T children studied, has been displayed in Table 3.

**TABLE 3:** Findings detected in the abdominal cavity, peripheral lymph node, and lung ultrasound (US) in ataxia-telangiectasia (A-T) children.

Results of US examinations in A-T children					

Patient	US examinations at follow-up visits (every 8–12 mo)	US of the abdominal cavity (echostructure of parenchymal organs)	US of the abdominal cavity (mesenteric and visceral lymph nodes)	US of cervical and submandibular lymph nodes	Lung US
Pt 1	1	Normal	Enlarged, heterogeneous mesenteric nodes	Enlarged, normoechogenic	
	2	Hepatomegaly	Enlarged, heterogeneous mesenteric nodes	Enlarged, normoechogenic	
Pt 2	1	Enlarged pancreas	Normal	Normal	B-line artifacts, consolidations
	2	Normal	Normal	Normal	
	3	Normal	Normal	Normal	
	4	Normal	Enlarged, hypoechogenic paraaortic nodes	Enlarged hypoechogenic cervical nodes	
Pt 3	1	Normal	Normal	Normal	
	2	Hepatomegaly	Normal	Normal	
	3	Hepatomegaly, hypoechogenic lesions in the spleen	Normal	Normal	
Pt 4	1	Hepatomegaly, hypoechogenic lesions in the liver	Enlarged visceral nodes in the liver hilum	Normal	
	2	Hepatomegaly, hypoechogenic lesions in the liver	Enlarged visceral nodes in the liver hilum	Normal	
	3	Hepatomegaly, hypoechogenic lesions in the liver	Enlarged visceral nodes in the liver hilum	Normal	
Pt 5	1–9	Normal	Normal	Normal	B-line artifacts
	10	Hypoechogenic focal lesions in the liver	Normal	Normal	
Pt 6	1	Normal	Normal	Normal	Normal
	2	Normal	Normal	Normal	B-line artifacts, consolidations
	3	Normal	Enlarged mesenteric and visceral nodes	Enlarged, normoechogenic cervical and submandibular nodes	B-line artifacts, consolidations, pleural effusion
	4–13	Hepatosplenomegaly, hypoechogenic lesions in the liver and spleen	Enlarged mesenteric and visceral nodes	Enlarged, normoechogenic cervical and submandibular nodes	B-line artifacts, consolidations, pleural effusion
Pt 7	1	Normal	Normal	Normal	
Pt 8	1	Normal	Normal	Normal	
Pt 9	1–4	Normal	Normal	Enlarged, normoechogenic submandibular nodes	
Pt 10	1–4	Normal	Normal	Normal	Normal
	5–10	Normal	Enlarged, normoechogenic mesenteric nodes	Enlarged, normoechogenic cervical and submandibular nodes	B-line artifacts, consolidations, pleural effusion
Pt 11	1–2	Normal	Normal	Enlarged, hypoechogenic cervical and submandibular nodes	B-line artifacts, consolidations

### Radiographic examinations

In 6 children, before establishing the definitive A-T diagnosis, chest X-rays (CXR) were performed due to the suspicion of pneumonia. In 5 of them, consolidations characteristic of respiratory tract infection were detected. Either sign of atelectasis or emphysema were not observable, yet in one patient CXR image suggested bronchiectasis. No chest CT examinations were conducted due to the need for extended diagnostics and instead, lung MRI was proposed.

### Magnetic resonance assessment

**FIGURE 3:**
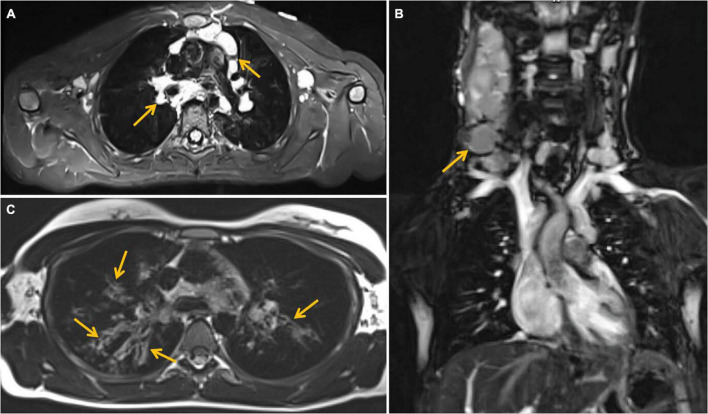
Magnetic resonance imaging (MRI) examination of the chest. **(A)** T2 -weighted Short-TI Inversion Recovery (STIR) axial image showing enlarged perivascular lymph nodes (marked with arrows), **(B)** coronal image—enlarged cervical lymph nodes (marked with an arrow). **(C)** T2-weighted blade axial image—bronchiectasis (marked with arrows) in upper lobes of both lungs, more severe on the right side.

**FIGURE 4:**
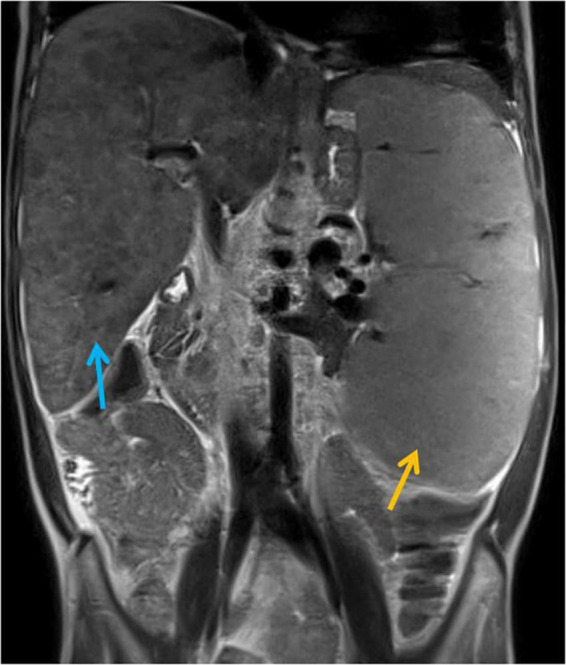
Magnetic Resonance Imaging (MRI) examination of the abdominal cavity, T2-weighted coronal image. Massive hepatosplenomegaly (the liver and spleen are marked with blue and orange arrows, respectively) and granulomatous lesions in the liver and spleen resulting in severe portal hypertension, hypersplenism, and ultimately, hepato-renal syndrome.

The efficacy of lung MRI has been increasingly highlighted due to the need for special ionizing radiation-free care that patients with IEI and defective DNA reparation should receive. In the study group, MRI revealed lymphatic nodal masses in the mediastinum, airway lesions characteristic of bronchiectasis, as well as parenchymal consolidations. Moreover, in the female patient with abnormal lymph node image of the neck, MRI enabled the assessment of their enhancement after contrast dose administration. MR images of lymphadenopathy and bronchiectasis are shown in Figures 3A–C. The diagnostic monitoring protocol also included MRI in A-T patients to monitor the liver and spleen pathology. The findings detected in MRI examinations in our A-T patients studied, are summarized in Table 4. The MRI examination of the brain was performed on one A-T patient with NHL and it did not reveal any abnormal imaging features. The majority of the examinations in children affected with A-T were performed without sedation on free or held breath. One A-T patient with the end-stage of the disease, chronic respiratory failure, and abdominal organomegaly, in whom MRI was made because of granulomatous lesions in the abdomen, which is displayed in Figure 4, required an anesthesiologist’s assistance.

**TABLE 4:** Findings detected in magnetic resonance imaging (MRI) examinations of the abdominal cavity and the chest in ataxia-telangiectasia (A-T) patients and the time of observation.

MRI examinations				

Patients	MRI of the chest		MRI of the abdominal cavity	
Pt 2	4th year	Enlarged cervical, infraclavicular and mediastinal lymph nodes, non-Hodgkin lymphoma		
Pt 5	6th year	Bronchiectasis		
Pt 6	3rd year	Bronchiectasis	3rd Year	Hepatosplenomegaly, focal granulomatous lesions in spleen and liver

## Discussion

While protection against ionizing radiation and the use of lung US has been underscored in children with DNA reparation defects, the use of this diagnostic technique is increasingly proposed in the analysis of respiratory tract pathologies in pediatric patients (18–20). Its usefulness in detecting and monitoring pneumonic consolidations and atelectasis, an alveolar-interstitial syndrome that may correlate with interstitial lung disease in IEI or pulmonary fibrosis, pleural effusion, as well as diaphragmatic excursion has been documented (21–24). The lung US findings correlated with pulmonary symptoms supporting the role of US imaging in monitoring lung disease in A-T and supporting the significance and predictive role of this imaging technique in clinical pathology. The lung US is therefore a promising imaging technique in A-T patients requiring regular follow-up examinations, being a convenient, inexpensive, available at the bedside, and safe ionizing radiation-free choice. However, like all US examinations, its outcome depends on the skills and experience of the physician as well as the quality of the device.

Consistently with results of other reports (5, 6, 25), indicating that A-T patients are affected with chronic inflammatory non-alcoholic fatty liver disease, a high rate of hepatomegaly, with concomitant focal lesions in the liver and/or spleen was noted in our pediatric study group. The impaired nuclear and cellular ATM kinase activity, with defective DNA double-strand break reparation, oxygen species production, and disturbed protein homeostasis are hypothesized to contribute to the organ-specific immunopathology in the liver. Systemic granulomatosis involving the liver and the spleen as a unique form of A-T-related granulomatous disease has also been reported in one of our patients (9, 11). US evaluation of peripheral lymph nodes using a basic examination technique (26) and sonoelastography and CD (27) is helpful in distinguishing between benign and malignant lymphadenopathy. The latter options have been reported to show very good sensitivity and specificity in detecting and monitoring lymph node malignancies and thereby their eligibility in routine monitoring of cervical lymphadenopathy in children has been defined (27, 28). Concerning US features for malignancy may include heterogeneity of the node, round shape as opposed to a normal oval shape, narrow or absent hilum, irregular borders, cystic necrosis, or irregular blood flow to the capsule (29, 30). Radiologists could also guide pediatricians in the differential diagnosis of peripheral lymphadenopathy, discrimination between its infectious, such as EBV, *Cytomegalovirus*, *Parvovirus B19*, *Toxoplasma gondii*, *Bartonella henselae*, *Mycobacterium tuberculosis*, and non-infectious causes, for example, Kawasaki disease, autoimmune lymphoproliferative syndrome, Kikuchi-Fujimoto, and Castleman disease, as well as malignant disorders, such as NHL (31–33). Whereas in most cases, lymphadenopathy is benign in its nature, watchful observation and a clinical examination with an US evaluation are required, while MRI imaging is advocated primarily in preparation for possible surgical interventions (29, 34).

The lung US examination has emerged as a complementary imaging technique to high-resolution computer tomography (HRCT), being of utmost importance in DNA reparation defects due to the increased risk of malignancy, and proved to be a valuable and applicable method for the assessment of ILD (35, 36). Multiple, diffuse B-line artifacts representing the sonographic hallmarks of the pulmonary interstitial-alveolar syndrome were found in as many as 5 out of 11 A-T children studied. Whereas ILD in IEI may occur in diverse clinical entities (37) and these conditions may share a similar B-line distribution pattern, lung US may play an adjuvant role when combined with clinical patient profile and aid in differentiating between cardiogenic and non-cardiogenic pulmonary edema, interstitial pneumonia, and pulmonary fibrosis (35, 36, 38). ILD is an inflammatory organ-specific immunopathology, characterized by multifactorial etiology, predominately related to immune dysregulation and hyperinflammation. The diagnosis of ILD in A-T patients is complex and includes clinical manifestations, such as cough and dyspnea, thoracic imaging findings, and/or surgical lung biopsy to indicate precisely the nature of possible etiologies of this condition. The pathogenesis of ILD in A-T patients is unknown, yet the role of respiratory viruses, *Mycoplasma pneumoniae*, and herpes viruses, for example, EBV, cytomegalovirus, and human herpes virus 6 (HHV6) infection in initiating and triggering the progression of chronic interstitial infiltrates and pulmonary fibrosis needs to be clarified (39–41). Among our 5 A-T children studied, who presented features of ILD in lung US examination, we have found EBV-DNA in peripheral blood and CMV-DNA was persistently present in 3, and HHV6 in one of them, reflecting a combined immunodeficiency (37, 40). It has been hypothesized that the immunodeficiency phenotype and lymphocyte dysregulation-driven hyperinflammation due to the lack of ATM activity in A-T are contributory factors to interstitial pneumopathy and lung fibrosis (42, 43).

Due to increasing concerns about potentially harmful effects on imaging techniques based on exposure to ionizing radiation in children, in particular in those affected with syndromic DNA reparation defects, MRI of the respiratory system plays an important role in diagnosing and monitoring a spectrum of thoracic disorders. MRI has been adopted for evaluation of the diseases in the lung parenchyma, interstitial lung disease, disorders of large as well as medium and small airways, abnormalities of thoracic vasculature, pleural diseases, masses, and chest wall pathologies (44–50). The role of MRI in neuroimaging and investigating neurodegeneration in A-T has also been recently defined in several reports (51, 52). With the ever-increasing technical improvements and number of indications in pediatric patients, the cooperation of pediatric radiologists and pediatric clinicians is required to define and delineate guidelines for MRI imaging feasibility and application in A-T (48, 51, 52).

Despite the relatively small number of participants due to the rarity of A-T which is a major limitation of this study, the use of modern US capabilities and MRI is efficient and recommended imaging diagnostic tool in monitoring children with IEI and DNA instability syndromes.

The radiologist’s approach provides an integral contribution to the multidisciplinary care of respiratory tract disorders in A-T patients. This also includes regular lung function monitoring aimed at early detection of lung disease progression and indicating the need for therapeutic interventions. Progressive neurodegeneration and wheelchair bounding lead to an inability to perform the required respiratory maneuvers during spirometry thereby making it difficult to obtain reliable reproducible lung function tests. For these A-T children who cannot manage to perform spirometry reliably, impulse oscillometry, measuring the airway resistance, is an alternative. Chronic lower airway infections and the hyperinflammatory state lead to bronchial wall damage and the development of bronchiectasis observable in MRI and manifesting as obstructive ventilation impairment. Difficulties in expiration due to muscle fatigue and atrophy, and chest wall deformity resulting in reduced tidal volume and restrictive ventilation disorders which may accompany imaging features of ILD and lung fibrosis (13, 15).

## Conclusion

In conclusion, while the analyzed patient group is affected with a severe IEI with susceptibility to ionizing radiation, it is characterized by the need for constant clinical supervision of the multidisciplinary team including an experienced pediatric radiologist and immunologist. The choice of an adequate, safe imaging diagnostic technique is crucial for the patient’s monitoring and, at the same time, this technique should provide the best answer to the clinician’s requests.

## Data availability statement

The raw data supporting the conclusions of this article will be made available by the authors, without undue reservation.

## Ethics statement

Ethical review and approval was not required for the study on human participants in accordance with the local legislation and institutional requirements. Written informed consent to participate in this study was provided by the participants’ legal guardian/next of kin.

## Author contributions

KJ-P was responsible for the design of the study, collection, and analysis of patients’ data, their interpretation, and drafted the initial manuscript. JP participated in patients’ data collection and analysis. AS-P was responsible for the clinical evaluation of patients, collection and security of patients data, participated in drafting the initial manuscript, and critically revised its final version. All authors contributed to the article and approved the submitted version.
